# Frequency and duration of sensory flicker control transcriptional profiles in 5xFAD mice

**DOI:** 10.1063/5.0261497

**Published:** 2025-07-08

**Authors:** Sara Bitarafan, Alyssa F. Pybus, Felix G. Rivera Moctezuma, Mohammad Adibi, Tina C. Franklin, Annabelle C. Singer, Levi B. Wood

**Affiliations:** 1 Parker H. Petit Institute for Bioengineering, Georgia Institute of Technology, Atlanta, Georgia, USA; 2 George W. Woodruff School of Mechanical Engineering, Georgia Institute of Technology, Atlanta, Georgia, USA; 3 Wallace H. Coulter Department of Biomedical Engineering, Emory University and Georgia Institute of Technology, Atlanta, Georgia, USA; 4 School of Biological Sciences, Georgia Institute of Technology, Atlanta, Georgia, USA

## Abstract

Current clinical trials are investigating gamma frequency sensory stimulation as a potential therapeutic strategy for Alzheimer's disease (AD); yet, we lack a comprehensive picture of the effects of this stimulation on multiple aspects of brain function. We previously showed that exposing mice to visual flickering stimulation increased mitogen activated protein kinase and nuclear factor kappa-light-chain-enhancer of activated B cells signaling in the visual cortex (VC) in a manner dependent on the duration and frequency of stimulation. Because these pathways control multiple neuronal and glial functions, here we aimed to define the transcriptional effects of different frequencies and durations of audiovisual flicker (AV flicker) stimulation on multiple brain functions. Within the VC, we found that all stimulation frequencies caused fast activation of a module of immune genes within 0.5 h and slower suppression of synaptic genes after 4 h. In the hippocampus, we found that a 20 Hz AV flicker activated a module of genes associated with mitochondrial function, metabolism, and synaptic translation, while 10 Hz rapidly suppressed a module of genes linked to neurotransmitter activity. Collectively, our data indicate that the frequency and duration of AV flicker stimulation control immune, neuronal, and metabolic genes in multiple regions of the brain affected by AD.

## INTRODUCTION

Pharmaceutical strategies targeting a single pathological hallmark of Alzheimer's disease (AD), such as amyloid beta (Aβ) accumulation have remained challenging, suggesting that successful treatment methods must target multiple key aspects of the disease.^1–5^ Multiple studies, including ours, have shown that 40 Hz visual and/or audio stimulation induced microglial changes associated with engulfment phenotypes with the ability to clear Aβ.^6–9^ Moreover, we have recently shown that exposing wild-type (WT) mice to different frequencies of visual stimulation (20 Hz, 40 Hz, random) elicited distinct patterns of activation of the nuclear factor kappa-light-chain-enhancer of activated B cells (NFκB) and mitogen activated protein kinase (MAPK) pathways.^6^ These pathways are central regulators of gene expression, cell survival, and immune function in AD.^10,11^ Thus, noninvasive sensory stimulation holds the potential to simultaneously affect multiple aspects of brain function with tunable effects depending on the frequency and duration of stimulation, which may provide essential utility for treating multifactorial diseases such as AD. Yet, we lack a comprehensive understanding of the effects of different frequencies and durations of combined audiovisual flicker (AV flicker) on brain function in the context of AD pathology. Given the apparent multi-potency of AV flicker stimulation, the goal of the current study was to utilize transcriptional profiling to holistically define the effects of differing frequencies and durations of AV flicker stimulation on a multitude of brain functions in a mouse model relevant to AD.

Therapeutic avenues for AD need to address multiple aspects of tissue pathology, going beyond the traditional pathological hallmarks, such as Aβ accumulation and neurofibrillary tangles. New strategies should target dysregulation of tissue metabolism, microglial-mediated immune dysfunction, impaired astrocyte support function, and reduced neurogenesis, among others.^12,13^ Collectively, the prior body of work around audio and/or visual sensory stimulation shows that it has the capability to change important aspects of brain function in multiple settings.^6–9,14^ Signaling pathways modulated by AV flicker stimulation, including the NFκB and MAPK pathways, control numerous aspects of brain function, such as neuronal survival, synaptogenesis, microglial activation and functional state, and astrocyte reactivity, among many others.^11,15,16^ We previously showed that specific durations of flicker stimulation induce phosphorylation of these pathways, while different frequencies of flicker have different effects on immune-modulatory cytokine expression.^6^ Specifically, 40 Hz flicker rapidly (∼5–15 min) activates neuronal signaling within MAPK and NFκB pathways, and 1 h of 40 Hz flicker stimulation triggers increased expression of cytokines, which ultimately yields gene expression alterations. Although NFκB and MAPK pathways are master regulators of downstream gene expression that control diverse cellular functions in the brain, there remains a gap in knowledge on how different frequencies and durations of AV flicker modulate gene expression related to different neuronal and glial functions. Moreover, current clinical trials in AD patients have demonstrated that 1 h per day of sensory stimulation can induce protein changes in the cerebrospinal fluid.^7,17,18^ Given that cell signaling and gene expression are dynamic processes,^19^ we used varying durations of AV flicker ranging from 30 min to 4 h to capture both early and sustained gene expression responses that may parallel the dynamics observed in clinical studies.

Here, we sought to holistically define the potential multi-potency of AV flicker stimulation with different frequencies and durations of stimulation on gene expression profiles in the context of AD relevant pathology. While prior work has evaluated the effects of AV flicker on neural activity, any long-term effects would likely affect gene transcription. Therefore, we investigated how AV flicker affects gene transcription. To do so, we exposed the 5xFAD amyloidosis mouse model to a range of AV flicker frequencies (constant light (control), 10, 20, and 40 Hz) for durations of 0.5, 1, or 4 h, then used bulk RNA sequencing (RNAseq) to profile transcriptional changes in the visual cortex (VC) and hippocampus (HIP). We selected to use audiovisual stimulation rather than visual stimulation alone for this study because multimodal stimulation is shown to affect more brain regions, and prior studies report this stimulation modulates neural activity and AD pathology in the hippocampus.^14,20^ To gain a holistic and unbiased view of the transcriptional effects after stimulation, we used weighted gene co-expression network analysis (WGCNA)^21^ to identify modules of highly correlated genes. This dimensional reduction approach revealed that different frequencies and durations of stimulation distinctly affect neuron- and astrocyte-enriched gene modules and emphasizes the potential for AV flicker to target multiple brain cells and brain functions important to AD. Our data, therefore, support that targeted AV flicker therapies may be designed to stimulate gene expression changes related to these functions and provide a new multipotent avenue of AD therapeutics.

## RESULTS

### 40 Hz AV flicker increased immune-related gene expression compared to 20 Hz AV flicker in 5xFAD mice

**FIG. 1.**
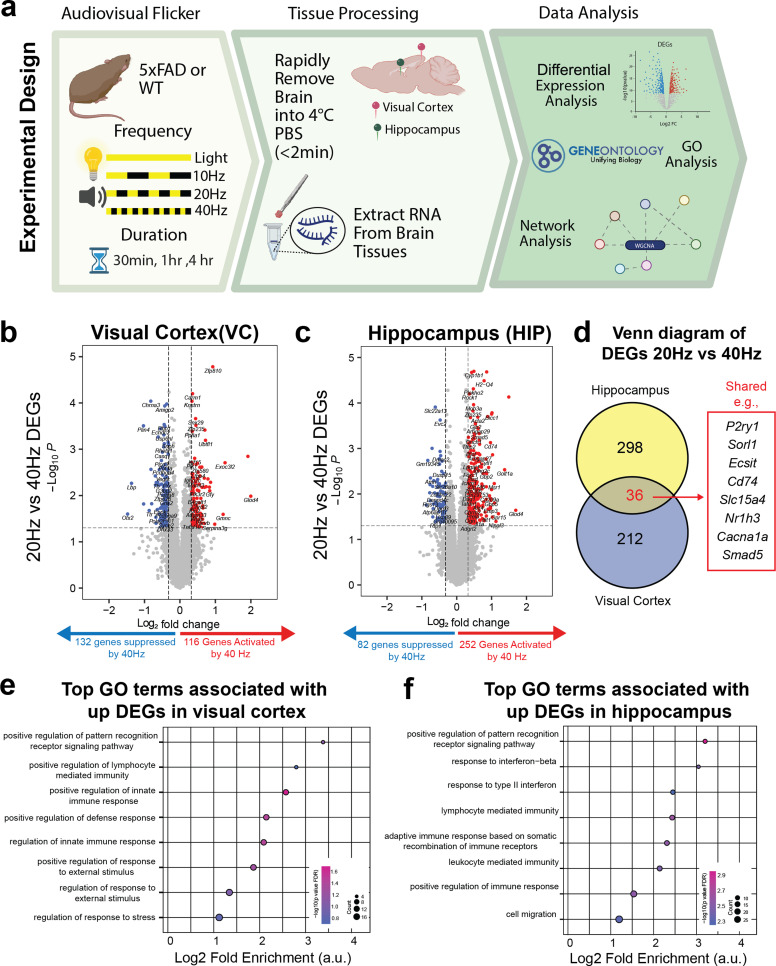
1 h of 40 Hz AV flicker increased immune-related gene expression compared to 20 Hz AV flicker in visual cortex and hippocampus of 5xFAD mice. (a) Experimental design for audiovisual stimulation. After stimulation, the visual cortices and hippocampi were isolated and sequenced via bulk RNA sequencing followed by statistical analysis (see Table S1 for sample sizes). (b) 1 h of 40 Hz or 20 Hz stimulation resulted in differentially expressed genes (DEGs) in the visual cortex and (c) hippocampus with upregulated DEGs in red and downregulated genes in blue (|log_2_ fold change| >0.25, p < 0.05). (d) Venn diagram of DEGs shared across regions comparing 20 vs 40 Hz. (e) 40 Hz gene ontology analysis of VC upregulated DEGs in 40 Hz identified significant GO terms associated with immune processes (FDR adjusted p < 0.05, dot size represents gene counts and dot colors indicate p-values). (f) GO analysis of HIP upregulated DEGs in 40 Hz identified significant terms associated with immune processes (FDR adjusted p < 0.05, dot size represents gene counts and dot colors indicate p-values). Panel A was created in BioRender. Wood (2025). https://BioRender.com/0r3cren.

Having previously found that 1 h of 40 Hz visual stimulation differentially activated immune signaling proteins compared to 20 Hz stimulation in male wild-type mice,^6^ we began the current study by asking if we could identify related transcriptional signatures in male 5xFAD mice exposed to 1 h of 40 or 20 Hz of AV flicker. We exposed male 5xFAD mice and wild-type (WT) littermates to 20 or 40 Hz AV flicker for 1 h, then conducted bulk RNAseq of the visual cortex (VC) and hippocampus (HIP) [Fig. 1(a), Methods, Table S1]. Of the 11, 312 genes remaining after filtering (Methods), we identified 248 differentially expressed genes (DEGs) in the visual cortex (132 upregulated in 20 Hz and 116 upregulated in 40 Hz) and 334 DEGs (82 upregulated in 20 Hz and 252 upregulated in 40 Hz) in the hippocampus [Figs. 1(b) and 1(c), and Table S2]. To gain insight into the functional implications of upregulated DEGs, we performed an overrepresentation gene ontology (GO) analysis, which revealed significant enrichment of immune-related GO terms in both regions [Figs. 1(d) and 1(e)]. While only 36 DEGs (including *Slc7a1*, *P2ry1*, and *Smad5*, Table S2) were shared across both regions, many of the biological processes enriched in 40 Hz in both regions were linked to immune processes (Table S3). The same analysis on tissues from WT littermates^6^ also identified DEGs in both brain regions (Fig. S1 and Table S4) but did not identify any significant GO terms. Nevertheless, our findings in 5xFAD mice indicate that 40 Hz AV flicker stimulation may enhance brain immune signaling in the context of amyloid beta pathology. These transcriptional changes are consistent with prior studies showing that 40 Hz flicker alters microglial function and signaling and further show that immune genes in the hippocampus are activated after just 1 h of stimulation.

### All AV flicker frequencies stimulated rapid activation of neuroimmune genes and slow suppression of synaptic genes in the visual cortex

**FIG. 2.**
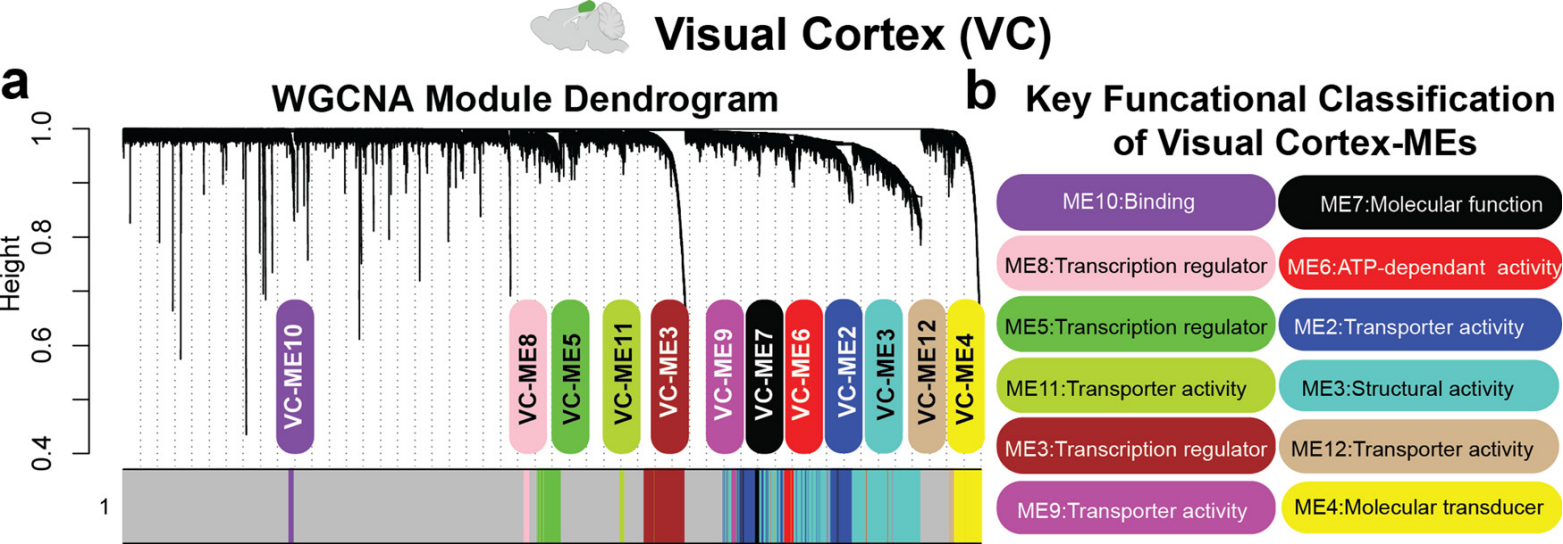
Visual cortex WGCNA identifies modules of co-expressed genes associated with frequency and duration of stimulation. (a) WGCNA identified 12 module eigengenes (MEs) in VC, with each color indicating a different ME. (b) Key functional classification of each module was identified via GO functional classification.

To evaluate if there were patterns of genes that similarly respond to all frequencies of stimulation in the visual cortex, we employed weighted gene co-expression network analysis (WGCNA) to identify modules of co-expressed genes that were highly correlated across frequency and duration of stimulation (Methods). WGCNA identified 12 module eigengenes (MEs, i.e., the first principal component of a given module representing the gene expression profile of highly co-varying genes within in a module) in the visual cortex [Fig. 2(a)], enabling us to collapse the 11, 312 genes into 12 modules of highly correlated genes. For genes within each WGCNA module, GO functional annotation (Table S5, Methods) revealed a diverse major functional classification among MEs [Fig. 2(b)]. The normalized z-score of each module eigengene (ME) was computed to compare across samples (Figs. S2A and S3). The heatmap of all z-scored module eigengenes suggested differences in the ME score patterns in response to different frequencies or durations of stimulation (Fig. S2A). Next, referencing previously published cell type-enriched mouse brain transcriptomes,^22,23^ we computed percent enrichment scores of genes within each ME, with high specificity in neurons, microglia, astrocytes, oligodendrocytes, and endothelia (Fig. S2B, Methods). Cell type percent enrichment indicates the fraction of co-varying genes from each cell type that comprise the ME and is suggestive of the main cell types represented by each ME. For example, Visual Cortex-ME9 was composed of a high percentage enrichment of astrocytic genes, while Visual Cortex-ME6 demonstrated high percent enrichment in neuronal genes (Fig. S2B). Finally, to ensure that the age of the mice was not a driver of the transcriptional signatures related to AV flicker, we correlated age with all identified modules (Fig. S4). Of those modules we report to be affected by AV flicker, only the neuron-enriched gene module Visual Cortex-ME6 had a significant correlation with age, as discussed below. We also applied WGCNA to age-adjusted gene expression data and found minimal effects on network composition (Fig. S5, Table S6).

**FIG. 3.**
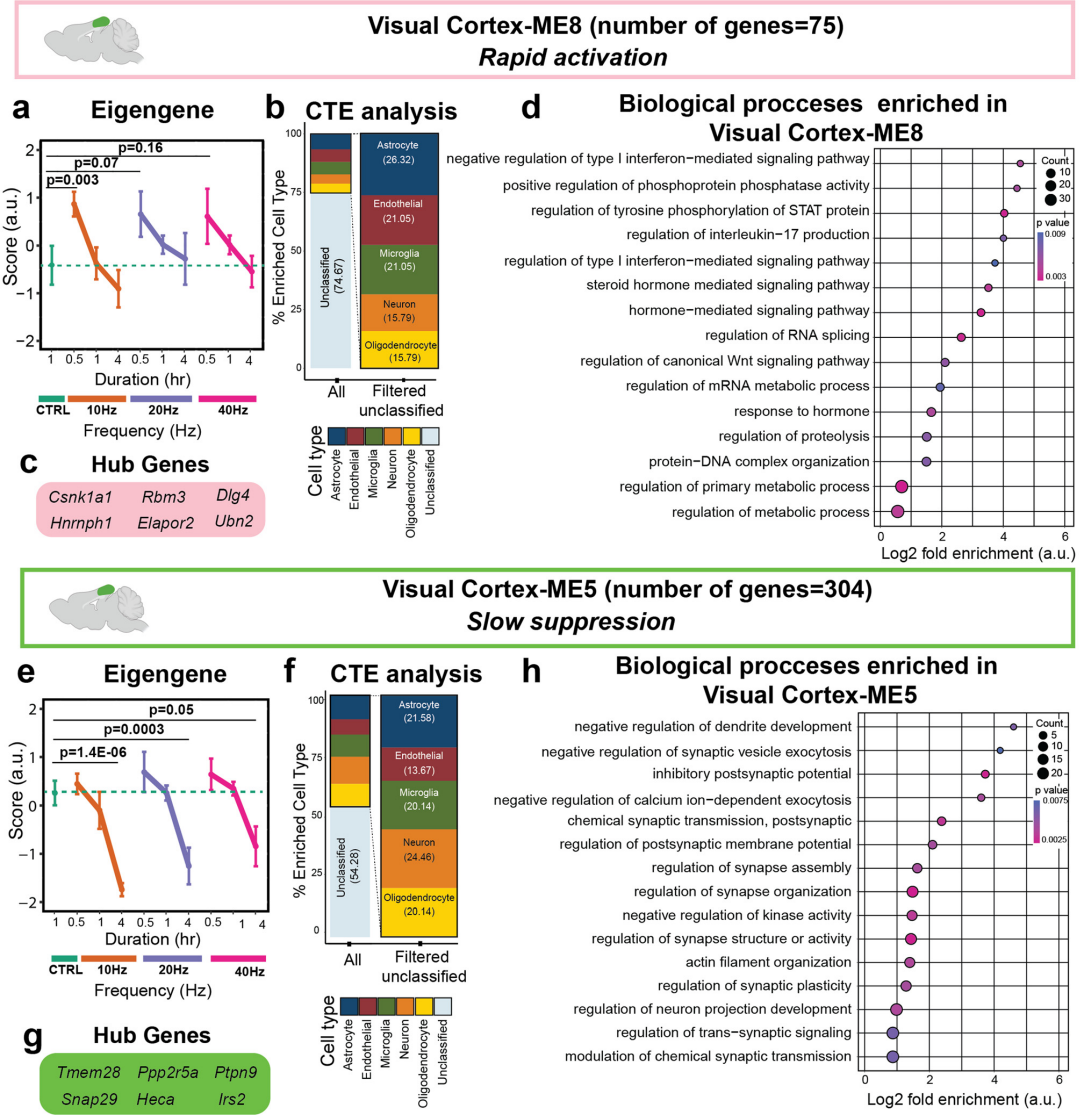
All frequencies of AV flicker drive rapid activation of neuroimmune genes and slower modulation of synaptic genes in the visual cortex. (a) Visual Cortex-ME8 (pink) genes demonstrated rapid activation in response to all frequencies of stimulation compared to the control group (sample sizes in Table S1, mean ± SEM, linear mixed model, Table S7). (b) Cell type percent enrichment (CTE) (Methods) revealed similar percent enrichment in Visual Cortex-ME8 genes across all cell types. (b) The six most highly correlated genes in Visual Cortex-ME8 were defined as hub genes. (d) Gene ontology enrichment of biological processes for Visual Cortex-ME8 genes (Fisher's exact test p < 0.05) identified significantly enriched immune-related GO terms associated with this module (dot size represents gene counts and dot colors indicate p-values). (e) All frequencies of AV flicker resulted in the slow suppression of Visual Cortex-ME5 (green) genes compared to the control group (sample sizes in Table S1, mean ± SEM, linear mixed model, Table S7). (f) Like Visual Cortex-ME8, Visual Cortex-ME5 did not demonstrate a notable percent enrichment in any of the five cell types assessed here. (g) The six most central genes in the module were determined as hub genes of Visual Cortex-ME5. (h) Gene ontology enrichment of biological processes for Visual Cortex-ME5 genes (Fisher's exact test p < 0.05) identified synaptic and neurodevelopment GO terms associated with this module (dot size represents gene counts and dot colors indicate p-values).

Among all visual cortex MEs, Visual Cortex-ME8 and Visual Cortex-ME5 genes showed consistent changes with the duration of AV flicker, regardless of frequency (Figs. 3 and S3). Visual Cortex-ME8 genes were rapidly upregulated after exposure to any frequency of AV flicker, then subsided back to control level by 4 h [Fig. 3(a)]. This module did not show notable percent enrichment for any of the cell types assessed in this study [Fig. 3(b)]. Interestingly, hub genes of the Visual Cortex-ME8 [Fig. 3(c)], such as *Csnk1a1*, *Ubn2*, *Elapor2*, and *Dlg4*, are all involved in modulating disease progression in many neurodegenerative disease,^24–28^ including AD. GO analysis of genes comprising Visual Cortex-ME8 revealed terms involving the regulation of interleukin 17 (IL-17) and type I interferon (IFN)-mediated signaling [Fig. 3(d)], both of which are associated with brain immune function.^29–31^ Visual Cortex-ME8 genes were also significantly enriched for GO terms associated with hormone signaling and RNA splicing, suggesting a broader effect on genes involved in brain signaling and transcriptional machinery [Fig. 3(d)]. In contrast to the rapid activation of Visual Cortex-ME8 genes, Visual Cortex-ME5 genes showed a pattern of slow suppression over longer durations [Fig. 3(e)]. Like Visual Cortex-ME8 genes, Visual Cortex-ME5 genes also did not show notable percent enrichment for any of the cell types assessed in this study [Fig. 3(f)]. GO processes enriched in Visual Cortex-ME5 genes are mainly related to synaptic activity and dendrite development [Fig. 3(h)]. Visual Cortex-ME5 hub genes, including *Tmem29*, *Snap29*, and *Irs2*, are involved in controlling neuronal excitability, vesicle-mediated transport, and brain immune signaling, which are all dysregulated in AD^32–34^ [Fig. 3(g)]. Together, Visual Cortex-ME8 and Visual Cortex-ME5 module patterns indicate that all frequencies stimulate short-term immune-related gene expression and slowly suppress gene expression associated with synaptic activity.

### An astrocyte-enriched gene module is modulated by AV flicker in a frequency- and duration-dependent manner in the visual cortex

**FIG. 4.**
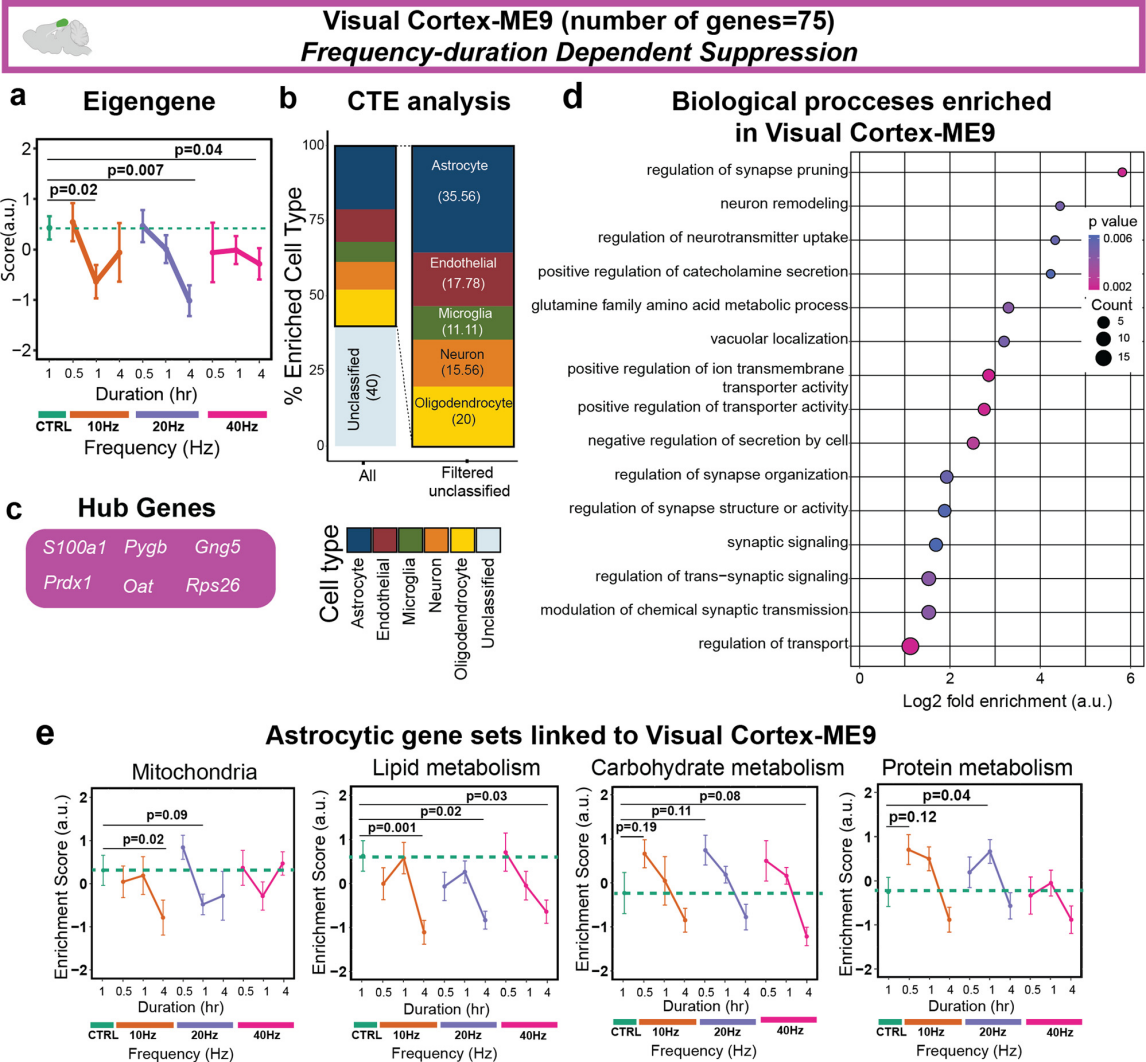
AV flicker frequency and duration both affect an astrocyte-enriched module of signaling-related genes in the visual cortex. (a) Visual Cortex-ME9 (magenta) demonstrated frequency- and duration-specific suppression in response to AV flicker (sample sizes in Table S1, mean ± SEM, linear mixed model, Table S7). (b) Astrocytic genes were the most prominent genes enriched in Visual Cortex-ME9. (c) The six most central genes in the module were determined as hub genes of Visual Cortex-ME9. (d) Gene ontology enrichment of biological processes for Visual Cortex-ME9 (Fisher's exact test p < 0.05) identified functions associated with synaptic signaling and neurotransmitter activity in this module (dot size represents gene counts and dot colors indicate p-values). (e) Gene set variation analysis of Visual Cortex-ME9 genes using custom cortical astrocyte gene sets (Methods) revealed frequency- and duration-dependent changes in astrocyte-specific functions in response to AV flicker.

Having found shared effects of AV flicker stimulation among all frequencies, we next looked for visual cortex MEs with distinct patterns of activation in response to different frequencies of AV flicker stimulation. Stimulation at all AV flicker frequencies suppressed Visual Cortex-ME9 genes at either 1 or 4 h of stimulation [Fig. 4(a)]. Interestingly, cell type percent enrichment analysis revealed that nearly 40% of Visual Cortex-ME9 genes were astrocyte enriched [Fig. 4(b)]. Astrocyte enrichment is consistent with the appearance of the astrocytic hub gene *S100a1* [Fig. 4(c)] and significantly enriched GO terms related to synaptic regulation, which is primarily mediated by astrocytes in the brain [Fig. 4(d)]. Because Visual Cortex-ME9 genes were astrocyte-enriched, we next assessed how different astrocyte-mediated functions were affected by AV flicker stimulation within Visual Cortex-ME9 genes by using gene set variation analysis (GSVA) together with our previously published astrocyte-enriched gene sets^35^ (Methods). Specifically, we computed enrichment scores of nine cortical astrocyte gene sets, including astrocytic lipid metabolism, astrocytic carbohydrate metabolism, astrocytic protein metabolism, and perisynaptic astrocyte processes (PAPs) [Figs. 4(e) and S6, and Table S8]. Interestingly, we found slow suppression of PAPs and lipid metabolism gene sets and rapid activation of carbohydrate metabolism gene sets in response to all frequencies of stimulation [Fig. 4(e)]. Collectively, astrocyte-enriched gene expression changes by AV flicker stimulation are regulated by both the duration and frequency of stimulation in the visual cortex.

### Different frequencies modulate genes related to neuronal functions in a duration-dependent manner in the visual cortex

**FIG. 5.**
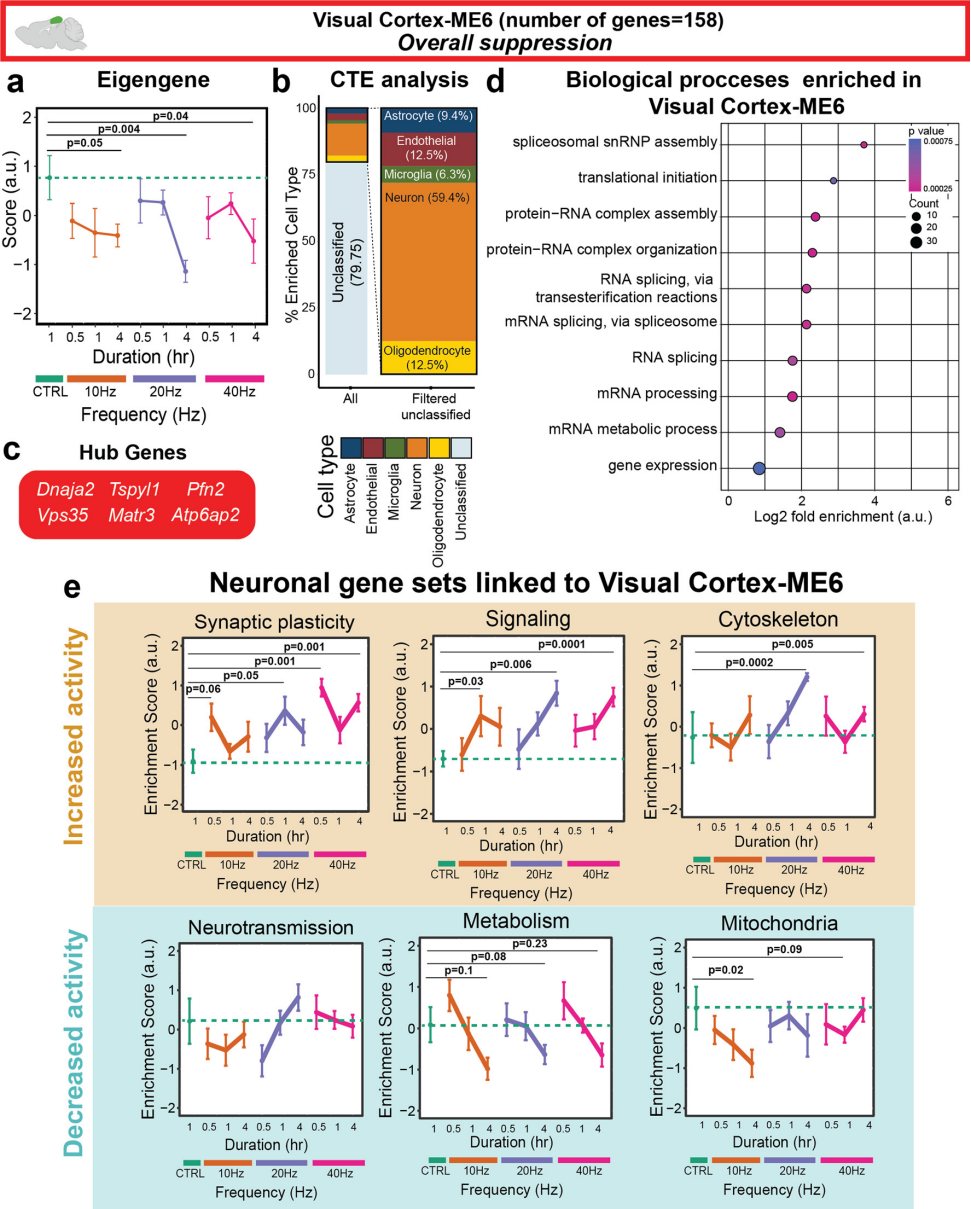
All frequencies of AV flicker suppress a neuron-enriched gene module and upregulate genes involved in neuronal signaling in a duration-dependent manner in visual cortex. (a) Visual Cortex-ME6 (Red) genes were suppressed by all frequencies of AV flicker in a duration-dependent manner (sample sizes in Table S1, mean ± SEM, linear mixed model, Table S7). (b) Neuronal genes were the most prominent genes enriched in Visual Cortex-ME6. (c) The six most central genes in the module were determined as hub genes of Visual Cortex-ME6. (d) Gene ontology enrichment of biological processes for Visual Cortex-ME6 (Fisher's exact test p < 0.05) identified transcriptional machinery related genes associated with this module (dot size represents gene counts and dot colors indicate p-values). (e) Gene set variation analysis of Visual Cortex-ME6 genes using custom cortical neuron gene sets (Methods) revealed all frequencies activated genes associated with synaptic plasticity, signaling, and cytoskeleton and suppressed neurotransmission, metabolism, and mitochondrial gene expression in a duration-dependent manner.

Since sensory flicker is believed to act via neurons, we concluded our visual cortex analysis by asking if we could identify a neuronally enriched gene signature that responded to AV flicker stimulation. Visual Cortex-ME6 genes were highly enriched (59.4% of classified genes) for neuronal genes and were suppressed across all frequencies [Figs. 5(a) and 5(b)]. As expected for a neuronally enriched module in 5xFAD mice, Visual Cortex-ME6 was correlated with age (R = −0.31, p = 0.0075). However, we found that the effects of AV flicker were greater than that of age and that the significant effects of AV flicker were maintained after adjustment for age (Fig. S7). Interestingly, hub genes of Visual Cortex-ME6 were mainly transcriptional regulators, such as *Dnaja2* and *Vsp35*,^36,37^ as well as nervous system development regulators, such as *Pfn2*.^38^ Of interest, *Dnaja2* is also shown to play a protective role against tau aggregation in AD^37,39,40^ and *Vsp35* has shown to be associated with late onset Parkinson disease.^41^ GO terms associated with Visual Cortex-ME6 genes were mainly involved in gene expression and transcription regulatory processes [Fig. 5(d)]. Like astrocytes, neurons are highly specialized cells. Therefore, to better understand how AV flicker affects neuron-enriched gene sets within Visual Cortex-ME6 genes (a neuron-enriched module), we used GSVA to compute enrichment scores for 14 neuronal gene sets from our prior work, including synaptic plasticity, signaling, neurotransmission, cytoskeleton, metabolism, and mitochondria (Methods). Interestingly, although AV flicker stimulation suppressed neuronally enriched genes in Visual Cortex-ME6, AV flicker led to enrichment of genes annotated for synaptic plasticity, signaling and cytoskeleton. AV flicker also suppressed metabolism and mitochondria gene sets within Visual Cortex-ME6 genes [Figs. 5(e) and S8, and Table S9]. Together, these findings suggest that while all frequencies of stimulation suppress genes involved in neuronal transcriptional machinery, genes controlling synaptic plasticity and signaling are enriched by all frequencies of stimulation in the visual cortex.

### Hippocampus exhibits slow suppression of a module enriched for neural differentiation, development, and signaling

We next asked if AV flicker stimulation induced frequency- and duration-specific effects in the hippocampus, a deep brain region affected early in AD. We conducted a separate WGCNA within the hippocampus (HIP), identifying 15 modules (Fig. S9, Table S10, Methods). Among all hippocampal MEs, Hippocampus-ME9, Hippocampus-ME12, Hippocampus-ME1, and Hippocampus-ME10 genes showed the most pronounced changes in response to AV flicker duration and frequency (Fig. S10). Of these MEs, none show significant correlation with age (Fig. S4), and we found that WGCNA based on age-adjusted gene expression data yielded similar network composition (Fig. S11).

**FIG. 6.**
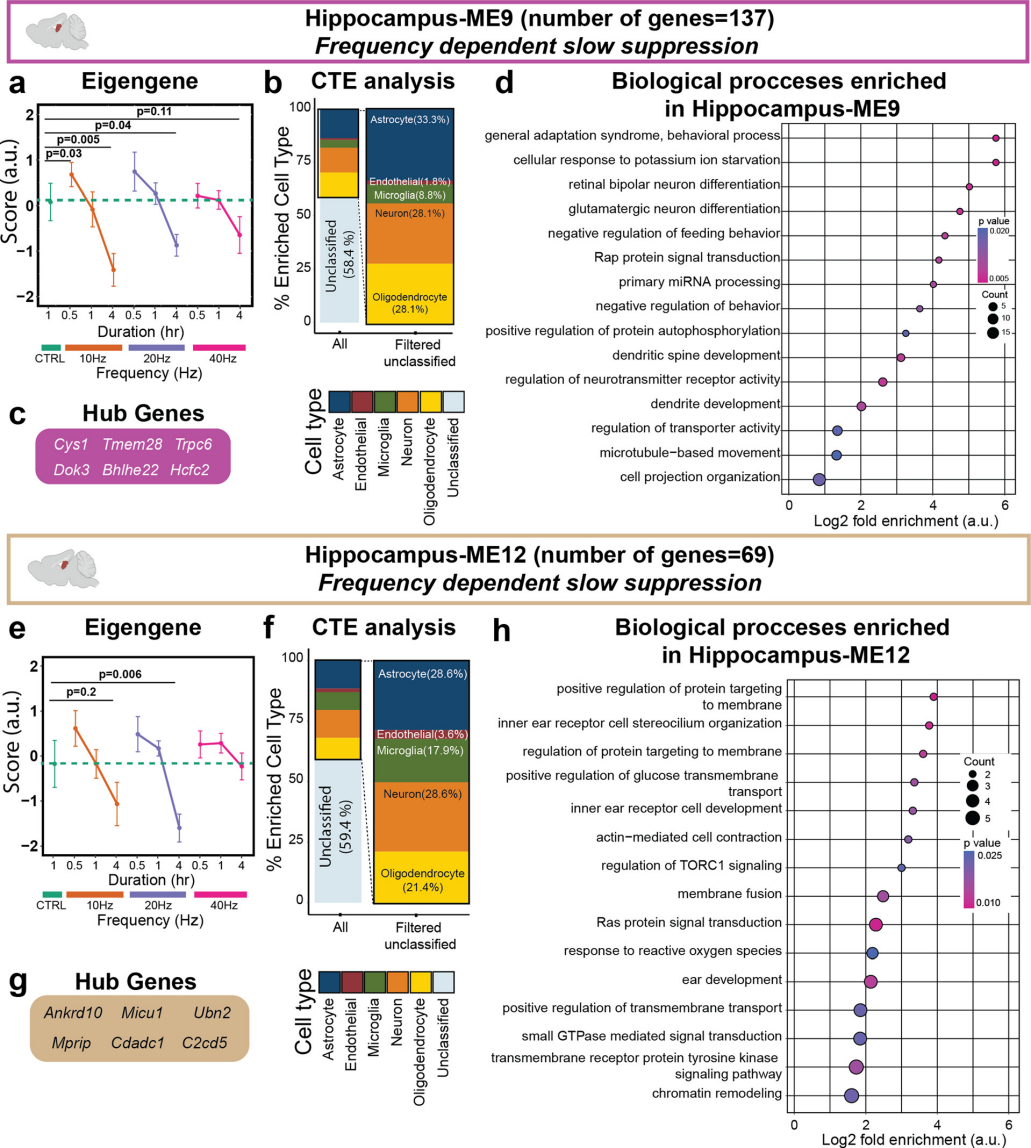
Prolonged 10 and 20 Hz AV flickers modulate gene modules enriched for neurotransmission, neural differentiation, and neuroimmune-signaling GO terms. (a) 10 and 20 Hz stimulations resulted in slow suppression of Hippocampus-ME9 genes (magenta) compared to the control (sample sizes in Table S1, mean ± SEM, linear mixed model, Table S7). (b) Hippocampus-ME9 CTE analysis showed percent enrichment for multiple cell types. (c) The six most central genes in the module were determined as hub genes of Hippocampus-ME9. (d) Gene ontology enrichment of biological processes for Hippocampus-ME9 genes (Fisher's exact test p < 0.05) identified enrichment of neurotransmission activity, neural differentiation, development, and behavior GO terms (dot size represents gene counts and dot colors indicate p-values). (e) 10 and 20 Hz stimulations resulted in the slow suppression of Hippocampus-ME12 (tan) genes compared to the control (sample sizes in Table S1, mean ± SEM, linear mixed model, Table S7). (f) CTE analysis showed high percent enrichment for multiple cell types within Hippocampus-ME12. (g) The six most central genes in the module were determined as hub genes of Hippocampus-ME12. (h) Gene ontology enrichment of biological processes (Fisher's exact test p < 0.05) identified signal transduction and neuroimmune changes associated within Hippocampus-ME12 genes (dot size represents gene counts and dot colors indicate p-values).

Genes in both Hippocampus-ME9 and Hippocampus-ME12 modules demonstrated slow suppression in response to 10 and 20 Hz of stimulation (Fig. 6). Despite the similar patterns of Hippocampus-ME9 and Hippocampus-ME12, GO analysis revealed that the genes comprising these modules control different functions. For example, 10 or 20 Hz frequency resulted in the slow suppression of Hippocampus-ME9 genes, which are enriched for genes associated with behavior, dendritic development, microtubule-based movement, neurotransmitter receptor regulation, and glutamatergic neuron differentiation [Figs. 6(a)–6(d)]. Hippocampus-ME12 genes also showed slow suppression after 10 or 20 Hz stimulation but were enriched for GO terms involving receptor tyrosine kinase (RTK) signaling pathway and response to oxygen species [Fig. 6(h)], all of which are processes dysregulated in AD.^42–45^ Neither of these modules demonstrated high enrichment to any of the five cell types assessed in this paper [Figs. 6(b) and 6(g)]. Together, these results show that a single session of prolonged AV flicker stimulation suppresses multiple hippocampal gene modules, each of which is associated with unique functions with frequency-specific effects.

### Hippocampal 10 Hz stimulation rapidly suppressed neuron-enriched gene module functions, while 20 Hz slowly activated genes involved in mitochondrial metabolism and synapse translation

**FIG. 7.**
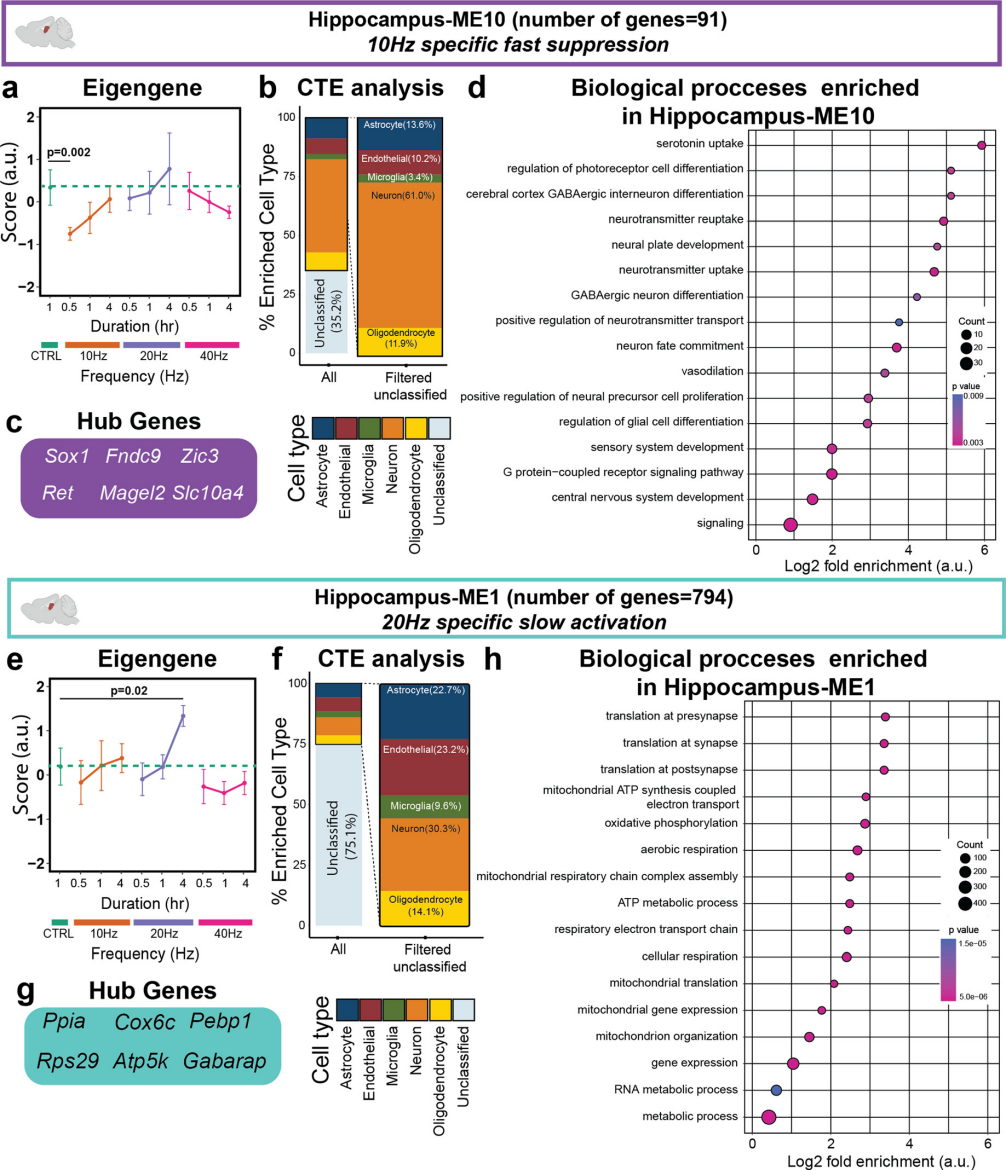
10 Hz AV flicker rapidly suppresses gene modules related to neurotransmission and differentiation, while 20 Hz slowly stimulates the gene module of mitochondrial and energy source genes. (a) 10 Hz stimulation rapidly suppressed Hippocampus-ME10 (purple) genes compared to the control (sample sizes in Table S1, mean ± SEM, linear mixed model, Table S7). (b) Neuronal genes were the most prominent genes enriched in Hippocampus-ME10 (∼60%). (c) The six most highly co-expressed genes in the module were determined as hub genes of Hippocampus-ME10. (d) Gene ontology enrichment of biological processes for Hippocampus-ME10 genes (Fisher's exact test p < 0.05) identified neurotransmitter regulation and neural differentiation as the main GO terms for Hippocampus-ME10 (dot size represents gene counts and dot colors indicate p-values). (e) 20 Hz stimulation slowly activated Hippocampus-ME1 (turquoise) genes relative to the control (sample sizes in Table S1, mean ± SEM, linear mixed model, Table S7). (f) CTE analysis showed high percent enrichment of multiple cell types assessed in this study. (g) The six most highly correlated genes in the module were determined as hub genes of Hippocampus-ME1. (h) Gene ontology enrichment of biological processes for ME1 genes (Fisher's exact test p < 0.05) identified synaptic translation, metabolism, and mitochondrial regulatory processes associated with the genes in Hippocampus-ME1 (dot size represents gene counts and dot colors indicate p-values).

In addition to the slow suppression effects of Hippocampus-MEs 9 and 12, AV flicker rapidly suppressed some hippocampus gene modules within 0.5 h of AV flicker in a frequency-dependent manner. Specifically, 10 Hz AV flicker rapidly suppressed the neuron-enriched Hippocampus-ME10 [Figs. 7(a) and 7(b)]. Hippocampus-ME10's hub genes were also neuronal-enriched, including *Fndc9*, *Slc10a4*, and *Sox1*, which are involved in nervous system development, neurotransmitter activity, and neural differentiation, respectively^46–48^ [Fig. 7(c)]. Although our custom gene sets annotated for cortical neurons did not reveal significant enrichment in the hippocampus, significant GO terms were associated with neurotransmission activity, neural development and differentiation, and neural signaling in Hippocampus-ME10 [Fig. 7(d)]. AV flicker also induced frequency-specific gene expression in the hippocampus. Specifically, 20 Hz AV flicker caused slow activation of Hippocampus-ME1 genes, which were enriched for GO terms associated with mitochondrial function, gene expression, and translation at synapses [Figs. 7(e)–7(h)]. Thus, AV flicker can be tuned based on frequency and duration to activate or suppress a distinct set of genes in the hippocampus.

**FIG. 8.**
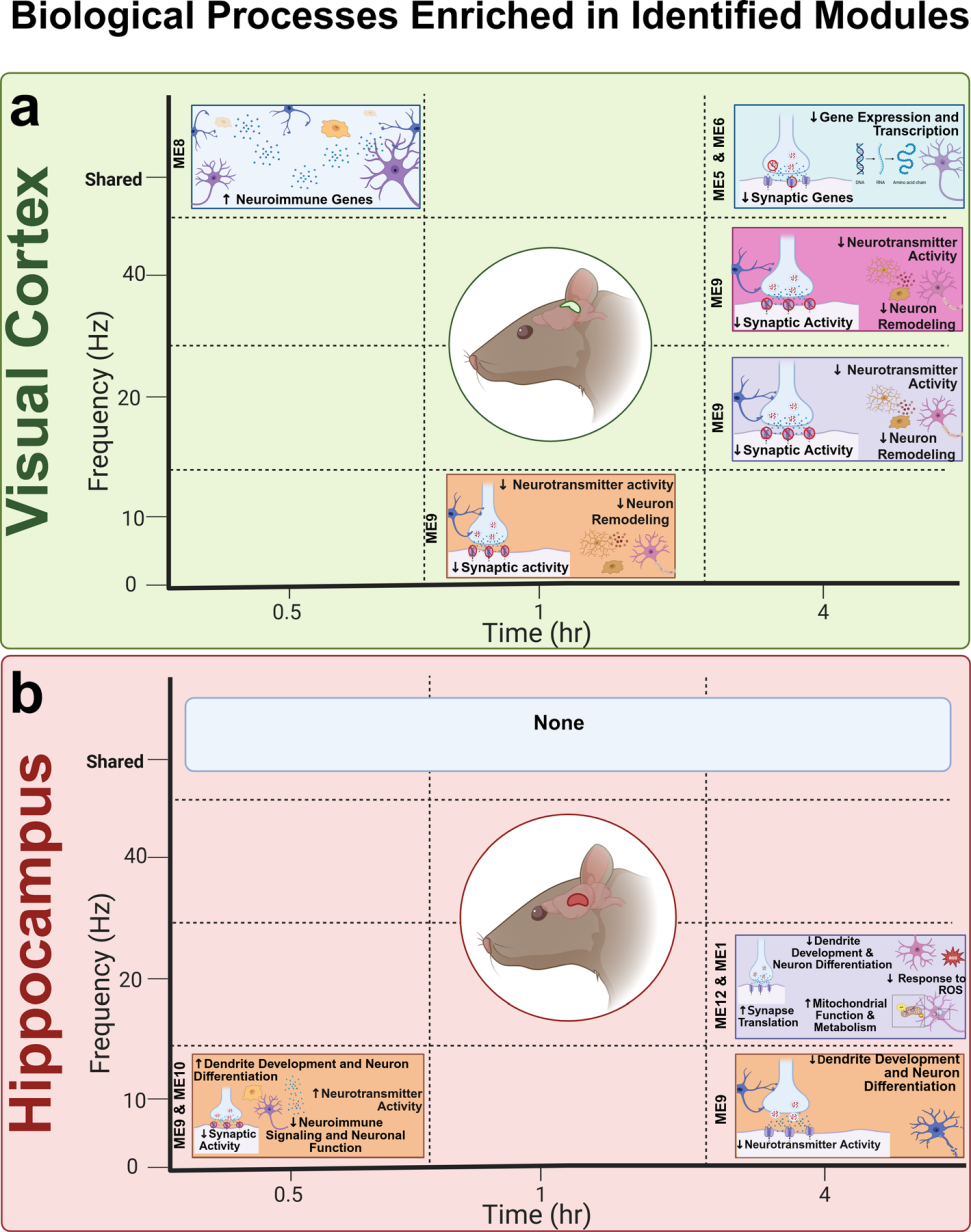
Map of stimulation parameter-induced transcriptional changes in the visual cortex and hippocampus. (a) Within the visual cortex, AV flicker stimulation drove transcriptional changes associated with neuroimmune, neuronal, and synaptic changes, with patterns that are either shared across all frequencies or specific to certain ones. The shared gene expression changes are associated with the upregulation of neuroimmune genes at 0.5 h and reduced synaptic function and gene expression at 4 h. The distinctive changes include reduced expression of genes involved in neurotransmission and neural remodeling after either 1 h of 10 Hz or 4 h of 20 Hz stimulation. Visual cortex MEs associated with each observed change are labeled with vertical text. (b) Within the hippocampus, AV flicker did not elicit shared transcriptional changes across frequencies at any time point. At 0.5 h of 10 Hz stimulation, genes involved in dendrite development, neurotransmitter activity, and neuronal differentiation processes were elevated, but genes involved in similar processes were decreased after 4 h of 10 Hz. Additionally, 4 h of 20 Hz stimulation induced transcriptional changes, including increased synapse translation, reduced dendrite development, and increased mitochondrial function and metabolism. Hippocampus-MEs associated with each observed change are labeled with vertical text. Created in BioRender. Wood (2025). https://BioRender.com/q2wukhe.

Although the hippocampus gene modules' changes in response to AV flicker stimulation are distinct from the visual cortex, gene annotations in modules from both regions share many functions, including neurotransmission, dendrite remodeling, and neuro-immunity (Fig. 8). Nevertheless, different frequencies of stimulation had specific effects in each region. We found that 40 Hz drives transcriptional changes linked to synaptic activity, neuronal function such as neurotransmission activity and neuron remodeling (Visual Cortex-ME9, Visual Cortex-ME5), and gene expression (Visual Cortex-ME6) in the visual cortex but did not identify any significant changes driven by 40 Hz stimulation in the hippocampus. Also, in the visual cortex, prolonged 20 Hz stimulation suppressed genes enriched in neuronal transcriptional machinery (Visual Cortex-ME6), while in the hippocampus, the same frequency and duration suppressed genes associated with neuroimmune signaling (Hippocampus-ME12) and activated genes related to metabolism and synaptic translation (Hippocampus-ME1). Furthermore, 10 Hz stimulation led to the rapid activation of genes involved in neuroimmune signaling in the visual cortex (Visual Cortex-ME8), but rapid suppression of genes linked to neurotransmitter activity and neuronal differentiation in the hippocampus (Hippocampus-ME10). Thus, our data collectively indicate that, while similar effects can be achieved in the visual cortex and hippocampus, the frequencies and durations of stimulation need to be tuned based on both the cellular function and the region of the brain being targeted.

## DISCUSSION

This study presents the most detailed analysis to date of the multipotent effects of multiple frequencies and durations of AV flicker stimulation in two brain regions of the 5xFAD amyloid mouse model of Alzheimer's disease. We hypothesized that distinct frequencies and durations of stimulation would differentially modulate gene expression relevant to the functions of neurons, astrocytes, and microglia. To test this hypothesis, we used the 5xFAD mouse model, commonly used in Alzheimer's research due to its rapid development of key disease features like amyloid plaques and neuronal loss, closely resembling human Alzheimer's pathology. Because 5xFAD mice develop cognitive deficits starting at 4 months,^49,50^ we used 3–7 months animals to evaluate the effects of AV flicker in the early symptomatic age range, which is relevant to clinical therapy. By simultaneously analyzing the effects of frequency and duration of AV flicker, this is the first study to pinpoint stimulation parameters (i.e., frequency and duration) that optimally modulate transcriptional signatures associated with either neuronal or glial functions. Our data highlight that the optimal stimulation protocol is determined based on the brain process and region to be targeted (Fig. 8).

Two key stimulation variables we examined in this study are frequency and duration. We used WGCNA to provide an unbiased summary of gene expression trends in our data, then assessed each identified gene module for enhanced expression of specific biological processes (gene ontology) and cell type percent enrichment analysis (CTE). Indeed, we identified a shared effect of frequency in the visual cortex, where all frequencies (10, 20, and 40 Hz) led to the fast activation of genes involved in neuroimmune processes (Visual Cortex-ME8), prolonged regulation of genes linked to synaptic activity and plasticity (Visual Cortex-ME5), and prolonged suppression of genes involved in neuronal transcriptional machinery (Visual Cortex-ME6). Within the hippocampus, we did not observe any shared effects among different frequencies, and many of the flicker-induced effects were frequency specific. For example, 10 Hz stimulation acutely suppressed modules of genes associated with neuron fate commitment and neurotransmitter uptake (Hippocampus-ME10), while 20 Hz stimulation led to the prolonged activation of genes involved in synaptic translation, mitochondrial function, and metabolism (Hippocampus-ME1). We found some of the modules affected by AV flicker stimulation across both brain regions were enriched in glial or neuronal genes. Specifically, genes in Visual Cortex-ME9 linked to synaptic pruning and activity were enriched in astrocytic genes, while genes in Visual Cortex-ME6 linked to transcriptional machinery and genes in Hippocampus-ME10 linked to neurotransmitter activity were profoundly enriched in neuronal genes.

An extensive body of prior work has investigated the effects of AV flicker in response to 1 h of stimulation. Prior studies have shown that 40 Hz flicker has multiple effects, including reducing amyloid beta levels, altering microglia and astrocyte morphology, increasing glymphatic clearance, and more.^6–9,20^ These prior studies primarily focused on the effects of 1 h of 40 Hz flicker or repeated exposures to 1 h of 40 Hz flicker. Additionally, our own prior work found that short durations of flicker, as little as 5–15 min, resulted in the activation of MAPK pathway signaling, which is known to affect downstream transcription in neuronal, synaptic, and immune pathways in a duration-dependent manner.^51,52^ Consistent with the known duration dependence of MAPK and NFκB, we found that different durations of the same frequency elicited different downstream transcriptional changes (Fig. 8). Within the hippocampus, a short duration (0.5 h) of 10 Hz stimulation activated modules of genes linked to dendrite development and neurotransmitter activity, while a longer duration at 10 Hz led to the suppression of a distinct set of genes that were linked to similar functions. In other cases, the different durations led to the differential expression of genes with distinct functional roles. For example, within the visual cortex, while prolonged (4 h) 20 Hz stimulation led to a reduction in the expression of a module of genes linked to synaptic activity (Visual Cortex-ME9), 0.5 h of 20 Hz led to the activation of a module of genes linked to neuroimmune activity (Visual Cortex-ME8). In addition, 1 h of 10 Hz stimulation led to the suppression of genes involved in neurotransmission activity and synaptic function within astrocytes (Visual Cortex-ME9), while 4 h of 10 Hz stimulation led to the suppression of neuronal transcriptional machinery (Visual Cortex-ME6). Together, these findings emphasize the importance of the duration of stimulation in defining upstream signaling pathways and downstream transcriptional changes.

A key finding from our study is that AV flicker affects hippocampal gene expression in addition to the visual cortex, which is clinically important to target in AD. While prior studies have reported that 20 and 40 Hz visual stimulations for 1 h could induce molecular changes within the visual cortex, it was previously unknown whether or not just 1 h of audiovisual stimulation could modulate the hippocampus transcription profile. While our data show that AV flicker affects both regions, we found that the specific transcriptional changes are highly dependent on the region being targeted. Nevertheless, despite our observed differences in the two brain regions, we showed that each frequency or duration of AV flicker led to a specific transcriptional change in each region.

This study has several important limitations that necessitate future work. First, we limited the current work to males due to the known sexual dimorphism in 5xFAD mice and the need to be sufficiently powered across multiple frequencies and durations of stimulation. Second, we relied on bulk RNAseq to enable us to quantify transcriptomes across a total of 177 samples in two brain regions and two genotypes, but many of the pathways we identified are central regulators of cellular function in neurons, microglia, and other cell types. Our data support the effects of AV flicker on multiple major cell types and identify transcriptional changes enriched in different cell types. In future studies, single cell or single nucleus RNAseq will be essential to uncover how these pathways are becoming activated in specific cell types. Third, we used mRNA quantification to define flicker-induced changes in 5xFAD mouse brain, but there is limited concordance between mRNA and protein-level changes. For example, reduced gene expression may not immediately result in lower protein abundance due to factors including protein stability, translation efficiency, and post-translational modifications.^53^ Moreover, changes in expression can reflect a range of biological phenomena, from adaptive changes in cellular state to the loss or gain of specific cell populations. Therefore, proteomics and behavioral assays will be essential to assess the effect of AV flicker stimulation on tissue pathogenesis and function. Fourth, mice selected for this study had an age range from 3 to 7 months old due to collection constraints during the pandemic. To ensure the age of the mice was not a driver of the transcriptional signatures found here, we correlated age with all identified modules (Fig. S3) and adjusted Visual Cortex-ME6 for age. To ensure the robustness of our findings, we conducted additional WGCNA on age-adjusted data for both regions. Notably, this analysis yielded consistent results with all modules reported here, exhibiting identical patterns of module eigengene variation across conditions (Figs. S4 and S9, and Table S6). Because our primary objective was to understand how audiovisual flicker affects the brain already burdened by Aβ pathology, we generated our WGCNA model based on 5xFAD samples alone. Importantly, previous studies from our group and others have shown that flicker can indeed induce transcriptional changes in WT mice, prompting us to investigate the AV flicker effect in the presence of AD pathology. Nevertheless, because our results are drawn from 5xFAD mice, we note that our findings may be specific to the context of Aβ pathology. Although we identified important effects of AV flicker parameters (i.e., durations and frequencies), these detailed condition analyses should also be evaluated in wild-type mice and other neurological conditions in future work. Finally, one condition (20 Hz, 4 h) across both regions had a relatively small sample size (n = 3) due to failed RNA extraction and limitations with sample collection during the pandemic. Although we think this will have an overall limited effect on the network due to the large number of samples in the total study, results from those groups may be less robust.

## CONCLUSION

In summary, we found that transcriptional signatures, e.g., Visual Cortex-ME8, Visual Cortex-ME5, and Visual Cortex-ME6, are shared across all frequencies of stimulation, whereas others, e.g., Visual Cortex-ME9, Visual Cortex-ME6, Hippocampus-ME10, and Hippocampus-ME1, are specific to the frequency as well as the duration. These results highlight the importance of frequency, duration, and region in defining the effects of audiovisual stimulation in the context of AD pathology. We also demonstrated that gene modules targeted by AV flicker are linked to multiple functions that are affected in AD. Moreover, we demonstrated that different frequencies and durations modulate genes involved in multiple biological functions and cell types, which opens new avenues for novel noninvasive and multipotent treatment of AD. Collectively, our data indicate that the frequency and duration of AV flicker stimulation control immune, neuronal, and metabolic genes in multiple regions of the brain affected by AD. AV flicker stimulation may thus represent a potential therapeutic strategy that can be tuned based on the brain region and the specific cellular process to be modulated.

## METHODS

### AV flicker stimulation and sample collection

All animal work was approved by the Georgia Tech Institutional Animal Care and Use Committee. Male 3- to 7-month-old 5xFAD on a BL6/SJL background (RRID: MMRRC_034840-JAX) and wild-type (WT) littermates were used for this study. Mice were single-housed on a reverse 12-h light/12-h dark cycle. Animal housing rooms were equipped with a ventilation system that provides 12 air changes per hour, a temperature range of 64–79 °F, and 30%–70% relative humidity. Mice were habituated for 1 h in a cage before audiovisual stimulation. Mice were placed in enclosures designed for AV flicker exposure, consisting of three black opaque sides and one transparent side facing a strip of LEDs. Mice were subjected to LED lights flashing at different frequencies: 40 Hz (50% duty cycle: 12.5 ms on, 12.5 ms off), 20 Hz (50% duty cycle: 25 ms on, 25 ms off), 10 Hz (50% duty cycle: 50 ms on, 50 ms off), or constant light (control). The LED light intensity was approximately 400 lux at the head of the mouse. Audio stimulations were delivered at an intensity of 55–65 dB using a 10 Hz tone. The audio pulses were synchronized to visual stimulation and turned on and off periodically as described above (e.g., 40 Hz: 12.5 ms on, 12.5 ms off). These parameters are consistent with previous studies demonstrating changes in protein levels under similar parameters.^6,9,54^ These exposures lasted for either 0.5, 1, or 4 h. Following the stimulation exposure, mice were quickly anesthetized with 4% isoflurane, and within 3 min, they were decapitated. Their brains were then extracted, and the bilateral visual cortices and hippocampi were microdissected and flash-frozen using liquid nitrogen for subsequent RNA sequencing analysis (see Table S1 for sample size per group).

### RNA sequencing

RNA was isolated from visual cortices and hippocampi tissues using the Qiagen RNeasy kit (217804; Qiagen; Hilden, Germany) according to the manufacturer's protocols. Molecular Evolution core at the Georgia Institute of Technology sequenced paired end mRNA using NovaSeq 6000 Sequencing System to obtain a sequencing depth of 30–40 × 10^6^ reads per sample. Prior to sequencing, quality control was performed using a bioanalyzer to determine the RNA Integrity Number (RIN) of the samples (i.e., >7). A NEBNext Poly(A) mRNA Magnetic Isolation Module and a NEBNext Ultra II Directional RNA Library Prep Kit were used to prepare sequencing libraries. RNA alignment was performed by Molecular Research Lab (https://mrdnalab.com) and data were validated for FASTQ integrity and quality. Four technical replicates of each sample were merged, followed by using DNAstar ArrayStar. Next, Qseq reads were mapped using the mouse reference genome GRCm39 (GCF_000001635.27) from the reference database. For the read assignment, the threshold was set at 20 bp and 80% of the bases matching within each read. Duplicated reads were eliminated, and genes with less than 10 raw counts in at least 25% of the samples were removed from analysis. Non-coding genes were removed from the analysis. All gene counts were normalized using the *DESeq2* R package,^55^ available through Bioconductor. To adjust the data for age and batch effects by experiment day, we used a linear mixed model in the *removebatcheffect()* function from the *limma* R package,^56^ available through Bioconductor.

### Weighted gene co-expression network analysis

Weighted gene co-expression network analysis (WGCNA) was conducted in R using the *WCGNA*^21^ package. A WGCNA threshold power of 6 was chosen as it was the smallest threshold that resulted in a scale-free R^2^ value greater than 0.8. The network was constructed in a single block using *blockwiseModules()* using the following parameters: a power of 6, a minimum module size of 50, a maximum module size of 12 000, a deep split size of 4, a merge cut height of 0.15, a correlation type of bicor, and a “mean” TOM denominator. The score for each module was computed and visualized to represent the gene expression pattern associated with that module for each sample. The module eigengene (ME) is the first principal component of the gene expression matrix for all genes within a given module. It represents the most prominent underlying pattern of gene expression in that module, effectively summarizing the collective pattern of genes in that module. The eigengene score for each module serves as a single quantitative value that captures the overall expression trend of the module across samples. Significant differences in module eigengene scores between groups were assessed using linear mixed models constructed with the *limma* package in R, available through Bioconductor.

### Differential expression analysis

Differential expression analysis was conducted using the *limma* package in R. Frequency was used as a variable in the design matrix. Raw data were transformed and adjusted for both batch and age using a linear mixed model. Significance level (nominal p-value, linear model) and fold change (FC) were computed using the lmFit() function. Because a linear model was used, nominal p-values without adjustment were used, as described in Refs. 57–59. Differentially expressed genes (DEGs) were defined as those with |fold change| ≥0.25 and p < 0.05. DEGs were displayed using volcano plots using the *EnhancedVolcano* R package,^60^ available through Bioconductor.

### Gene ontology

Gene ontology was conducted for each of the MEs identified by WGCNA in both regions using the PANTHER overrepresentation test with PANTHER 18.0 through the Gene Ontology resource (https://geneontology.org/). The *Mus Musculus* GO biological processes complete annotation set was used with Fisher's exact test to compute the significance of the gene sets for each ME. For GO analysis of differentially expressed genes, Fisher's exact test followed by Benjamini–Hochberg false discovery rate (FDR) adjustment was applied. The reference list consisted of all detectable genes in our dataset after filtering. Major functional classifications of genes within each ME were identified using GO functional annotation through the Gene Ontology resource.

### Cell type percent enrichment analysis

Cell type percent enrichment analysis (CTE) was conducted using published cell type markers from five cell types within the brain (neuron, microglia, astrocyte, oligodendrocyte, and endothelia) from mouse brain proteome and transcriptome studies^22^ (Table S11). Briefly, for each module identified in WGCNA, percent enrichment was calculated by dividing the number of cell type-specific genes by the total number of genes in that module for each cell type across all MEs.

### Custom astrocyte and neuron gene set variation analysis

To study the functional impact of changes within specific cell types, gene set variation analysis (GSVA) was conducted, referencing previously published neuron or astrocyte custom gene sets.^35^ GSVA is an unsupervised enrichment algorithm that identifies variations of pathway activity by defining enrichment scores for gene sets, which each contain a set of genes that share the same cellular function. The *gsva* R package^61^ (available on Bioconductor) was used to identify the enrichment of custom-annotated neuron or astrocyte gene sets within neuron- or astrocyte-enriched module eigengenes identified by the cell type enrichment analysis.

### Data analysis and visualization

Data were analyzed and figures were generated in RStudio (Boston, MA, USA) using the R programming language. Figures were then further polished using Adobe Illustrator (San Jose, CA, USA) or BioRender (Toronto, ON, Canada). Software packages and functions described in this section are denoted by italic font. Heatmaps were generated using the R package *heatmap3*; line graphs and regression plots were created using the package *ggplot2*. Clustering was conducted using the *hclust* function of the stats package in R using Euclidean distance with Ward's D2 agglomeration method. Outlier detection within the transcriptomic data was conducted in R by calculating the Mahalanobis distance of each point from the centroid of the data within each region (α < 0.05). Two hippocampi and one visual cortex sample were detected as outliers and those samples were omitted prior to analysis. All module scores were z-scored. For statistical testing of modules and gene sets, we used the *limma* R package (available on Bioconductor) to build a linear mixed model to determine the group differences (control vs 10 or 20 or 40 Hz) between each module (WGCNA) or gene set (GSVA). For all analyses, unadjusted p < 0.05 was considered statistically significant. Confidence interval, FDR adjusted p-values, and Bonferroni adjusted p-values for MEs are reported in the corresponding supplementary material tables. All module scores were z-scored. Our goal in the current study was to capture even subtle gene expression changes in response to AV flicker stimulation. Because stringent multiple testing, such as the Bonferroni adjustment, is known to be overly conservative in high-dimensional datasets, its use could potentially obscure biologically meaningful gene expression patterns. Therefore, to balance the control for false positivity while maintaining sufficient statistical power to detect biologically meaningful patterns in gene expression affected by AV flicker, we employed a linear mixed model.

## SUPPLEMENTARY MATERIAL

See the supplementary material for the following: sample size of each experimental group (Table S1); cell type enrichment gene list (Table S11); DEGs_1hr_20 Hz vs 40 Hz stimulation in 5xFAD mice (Table S2); GO terms_DEGs_40 Hz vs 20 Hz of 5xFAD_VC_HIP (Table S3); DEGs_1hr_20 Hz vs 40 Hz_WT_VC_HIP (Table S4); GO terms associated with VC_MEs (Table S5); VC and HIP MEs in age-adjusted data (Table S6); summary of ME stats using the linear mixed model (Table S7); summary of VC_ME9 Custom GS_ Stats using the linear mixed model (Table S8); summary of VC_ME6 Custom GS_ Stats using the linear mixed model (Table S9); GO terms associated with HIP_MEs (Table S10); differentially expressed genes between 1 h of 20 and 40 Hz stimulation in WT mice (Fig. S1); WGCNA identifies 12 module eigengenes (MEs) in the visual cortex with different cell type enrichments (Fig. S2); WGCNA identifies 12 module eigengenes (MEs) in VC (Fig. S3); correlation heatmap between MEs and age (Fig. S4); WGCNA on age-adjusted VC gene expression data yielded consistent results with all modules reported, exhibiting identical patterns of ME variation across conditions (Fig. S5); custom astrocyte-specific gene sets associated with VC-ME9 (Fig. S6); VC-ME6 age adjustment (Fig. S7); custom neuron-specific gene sets associated with VC-ME6 (Fig. S8); hippocampus WGCNA identifies modules of co-expressed genes associated with frequency and duration of stimulation (Fig. S9); WGCNA identifies 15 module eigengenes (MEs) in HIP (Fig. S10); and WGCNA on age-adjusted HIP gene expression data yielded consistent results with all modules reported, exhibiting identical patterns of ME variation across conditions (Fig. S11).

## Data Availability

The data that support the findings of this study are openly available in Gene Expression Omnibus (GEO) repository under series record GSE255004 (https://www.ncbi.nlm.nih.gov/geo/query/acc.cgi?acc=GSE255004) Ref. [62].
